# Treadmill Slope Modulates Inflammation, Fiber Type Composition, Androgen, and Glucocorticoid Receptors in the Skeletal Muscle of Overtrained Mice

**DOI:** 10.3389/fimmu.2017.01378

**Published:** 2017-10-25

**Authors:** Alisson L. da Rocha, Bruno C. Pereira, Giovana R. Teixeira, Ana P. Pinto, Fabiani G. Frantz, Lucila L. K. Elias, Fábio S. Lira, José R. Pauli, Dennys E. Cintra, Eduardo R. Ropelle, Leandro P. de Moura, Rania A. Mekary, Ellen C. de Freitas, Adelino S. R. da Silva

**Affiliations:** ^1^Postgraduate Program in Rehabilitation and Functional Performance, Ribeirão Preto Medical School, University of São Paulo (USP), Ribeirão Preto, Brazil; ^2^Department of Physical Education, State University of São Paulo (UNESP), Presidente Prudente, Brazil; ^3^Department of Clinical, Toxicological, and Bromatological Analysis, Faculty of Pharmaceutical Sciences of Ribeirão Preto, University of São Paulo (USP), Ribeirão Preto, Brazil; ^4^Department of Physiology, Ribeirao Preto Medical School, University of São Paulo (USP), Ribeirão Preto, Brazil; ^5^Laboratory of Molecular Biology of Exercise (LaBMEx), School of Applied Sciences, University of Campinas (UNICAMP), Campinas, Brazil; ^6^Department of Pharmaceutical Business and Administrative Sciences, MCPHS University, Boston, MA, United States; ^7^Department of Surgery, Brigham and Women’s Hospital, Harvard Medical School, Boston, MA, United States; ^8^School of Physical Education and Sport of Ribeirão Preto, University of São Paulo (USP), Ribeirão Preto, Brazil

**Keywords:** overtraining, inflammatory signaling, skeletal muscle fiber type composition, androgen and glucocorticoid receptors

## Abstract

Overtraining (OT) may be defined as an imbalance between excessive training and adequate recovery period. Recently, a downhill running-based overtraining (OTR/down) protocol induced the nonfunctional overreaching state, which is defined as a performance decrement that may be associated with psychological and hormonal disruptions and promoted intramuscular and systemic inflammation. To discriminate the eccentric contraction effects on interleukin 1beta (IL-1β), IL-6, IL-10, IL-15, and SOCS-3, we compared the release of these cytokines in OTR/down with other two OT protocols with the same external load (i.e., the product between training intensity and volume), but performed in uphill (OTR/up) and without inclination (OTR). Also, we evaluated the effects of these OT models on the muscle morphology and fiber type composition, serum levels of fatigue markers and corticosterone, as well as androgen receptor (AR) and glucocorticoid receptor (GR) expressions. For extensor digitorum longus (EDL), OTR/down and OTR groups increased the cytokines and exhibited micro-injuries with polymorphonuclear infiltration. While OTR/down group increased the cytokines in soleus muscle, OTR/up group only increased IL-6. All OT groups presented micro-injuries with polymorphonuclear infiltration. In serum, while OTR/down and OTR/up protocols increased IL-1β, IL-6, and tumor necrosis factor alpha, OTR group increased IL-1β, IL-6, IL-15, and corticosterone. The type II fibers in EDL and soleus, total and phosphorylated AR levels in soleus, and total GR levels in EDL and soleus were differentially modulated by the OT protocols. In summary, the proinflammatory cytokines were more sensitive for OTR/down than for OTR/up and OTR. Also, the specific treadmill inclination of each OT model influenced most of the other evaluated parameters.

## Introduction

The enhancement and maintenance of performance during a competitive season are achieved by the completion of high intensity and volume exercise sessions. Considering these high-load exercise sessions may induce momentary performance decline and acute fatigue, the aforementioned optimal training adaptations depend on adequate recovery periods (1). Indeed, the imbalance between the process of intensified training [i.e., overtraining (OT)] and adequate recovery periods may lead to the nonfunctional overreaching (NFOR) state, which is characterized as a performance decline that may be linked to psychological and hormonal disruptions (2).

Regarding NFOR etiology, Smith introduced the cytokine hypothesis considering that OT induced musculoskeletal trauma, increasing the synthesis and release of interleukin 1beta (IL-1β), IL-6, and tumor necrosis factor alpha (TNF-α) (3, 4). These elevated serum proinflammatory cytokine levels would interact with different systems, initiating most of the signs and symptoms found in NFOR (2). Our research group corroborated the cytokine hypothesis (3, 4) showing high skeletal muscle levels of IL-6 and TNF-α as well as high serum levels of IL-6 after a downhill running-based OT (OTR/down) protocol (5).

Knowing that the increased responses of intramuscular and serum cytokine concentrations to acute and chronic eccentric exercise sessions such as downhill running are well established in the literature (6), we recently described the responses of IL-1β, IL-6, and TNF-α in whole gastrocnemius and serum samples after two other running OT protocols performed in uphill (OTR/up) and without inclination (OTR) (7). Approximately 45% of the sessions of OTR/down, OTR/up, and OTR were performed above the intensity corresponding to the maximal lactate steady state (7–11), which has been extensively used as a gold standard identifying the exercise intensity corresponding to the aerobic/anaerobic metabolic transition in rodents (12–17). Therefore, it is important to verify the cytokine contents in the skeletal muscle samples that are predominantly composed of type I and II fibers (18).

Herein, we first investigated the responses of proinflammatory cytokines in skeletal muscle samples predominantly composed of type I and II fibers. Based on the anti-inflammatory effects of IL-10 (19), the negative correlation between IL-15 and TNF-α (20), and the sensitivity of SOCS-3 to OTR/down (21), we also evaluated the release of these cytokines after the three OT models. Because skeletal muscle microtrauma reduces the range of motion (22) and strength (23), tissue injury may contribute to the NFOR state-induced performance decrease. Therefore, our second aim was to verify the effects of these OT protocols on skeletal muscle morphology and classical serum markers of muscle damage such as creatine kinase (CK) and lactate dehydrogenase (LDH) (24).

Because specific running slopes demanded different muscle recruitment patterns (25), our third aim was to investigate the pattern of skeletal muscle fiber type composition after these OT protocols. Considering the serum concentrations of IL-6 were upregulated after all OT models (7) and knowing that this cytokine was able to activate the hypothalamic–pituitary–adrenal (HPA) axis (26), we verified the serum concentrations of corti-costerone and the intramuscular expressions of the glucocorticoid receptor (GR) in overtrained mice. Based on the role of androgen receptor (AR) in skeletal muscle atrophy and weakness (27), we also investigated its expression.

## Materials and Methods

### Experimental Animals

Eight-week-old male C57BL/6 mice from the Central Animal Facility of the Ribeirão Preto campus were maintained in individual cages with controlled temperature (22 ± 2°C) on a 12:12-h light-dark inverted cycle with food (Purina chow) and water *ad libitum*. The experimental procedures were approved by the Ethics Committee of the University of Sao Paulo (ID 14.1.873.53.0). Rodents were randomly divided into control (CT; sedentary mice; *n* = 16), overtrained by downhill running (OTR/down; *n* = 16), overtrained by uphill running (OTR/up; *n* = 16), and overtrained by running without inclination (OTR; *n* = 16). Mice were manipulated and overtrained in a dark room between 6 and 8 am (28).

### Incremental Load Test

After being adapted to treadmill running (INSIGHT^®^, Ribeirão Preto, São Paulo, Brazil) for 5 days, 10 min day^−1^ at 3 m min^−1^, rodents performed the incremental load test with an initial intensity of 6 m min^−1^ at 0% with increasing increments of 3 m min^−1^ every 3 min until exhaustion, defined when mice touched the treadmill end 5 times in 1 min. Mice were encouraged using physical prodding, and when they became exhausted without completing the stage, the exhaustion velocity (EV; m min^−1^) was corrected according to Kuipers et al. (29). The EV of each mouse was used to prescribe the intensity of the OT protocols (5, 21, 28).

### OT Protocols and Performance Evaluations

Each experimental week of the 8-week running OT protocols performed in downhill, uphill, and without inclination consisted of 5 days of training followed by 2 days of recovery. The performance evaluations were applied on week 0 and 48 h after the last sessions of OT protocols at the end of weeks 4 and 8, and consisted of the rotarod test, incremental load test, exhaustive test, and grip force test (7, 9, 30–32). On week 0, the experimental groups performed the incremental load test without inclination. On the other hand, at the end of week 4 and 8, CT and OTR performed the incremental load test without inclination, OTR/down performed the incremental load test in downhill running, and OTR/up performed the incremental load test in the uphill running. The efficiency of these OT models in inducing NFOR state and the detailed description of the physical tests has been previously published by our group (7–9, 32).

### Skeletal Muscle and Total Blood Collections

Rodents were anesthetized 36 h after grip force test performed at the end of OT protocols. After a fast period of 6 h, mice were anesthetized with an intraperitoneal (i.p.) injection of 2-2-2-tribromoethanol 2.5% (10–20 µL g^−1^). As soon as anesthesia was confirmed by the loss of pedal reflexes, due to their different fiber type composition (18), extensor digitorum longus (EDL), and soleus muscles of both hindlimbs were removed and used for immunoblotting analysis or stored at −80°C for subsequent histological and immunofluorescence analyses. Subsequently, total blood was collected by decapitation, and serum was separated by centrifuging (1,100*g*) for 15 min at 4°C and stored at −80°C for subsequent determination of anti and proinflammatory cytokines, CK, LDH, and corticosterone.

### Immunoblotting Analysis

Extensor digitorum longus and soleus muscle samples were homogenized in extraction buffer (1% Triton X-100, 100 mM Tris, pH 7.4, containing 100 mM sodium pyrophosphate, 100 mM sodium fluoride, 10 mM EDTA, 10 mM sodium vanadate, 2 mM PMSF, and 0.1 mg mL^−1^ aprotinin) at 4°C with a Polytron PTA 20 S generator (Brinkmann Instruments model PT 10/35), operated at maximum speed for 30 s. The extracts were centrifuged (9,900*g*) for 40 min at 4°C to remove insoluble material, and the supernatants of these homogenates were used for protein quantification using the Bradford method (33). Proteins were denatured by boiling in Laemmli sample buffer containing 100 mM DTT, run on SDS-PAGE gel and transferred to nitrocellulose membranes (GE Healthcare, Hybond ECL, RPN303D). The transfer efficiency to nitrocellulose membranes was verified by brief staining of the blots with Ponceau red stain. These membranes were then blocked with Tris-buffered saline (TBS) containing 5% BSA, and 0.1% Tween-20, for 1 h, at 4°C.

Antibodies used for immunoblotting overnight at 4°C were IL-10 (SC52561), IL-15 (SC7889), suppressor of cytokine signaling 3/SOCS-3 (SC9023), and beta-actin (SC69879) from Santa Cruz Biotechnology (Santa Cruz, CA, USA); IL-1beta (AB9722) and IL-6 (AB6672) from Abcam (Cambridge, UK). After a wash with TBS containing 0.1% Tween-20, all membranes were incubated for 1 h at 4°C with secondary antibody conjugated with a horseradish peroxidase. The specific immunoreactive bands were detected by chemiluminescence (GE Healthcare, ECL Plus Western Blotting Detection System, RPN2132). Images were acquired by the C-DiGit™ Blot Scanner (LI-COR^R^, Lincoln, Nebraska, USA) and quantified using the software Image Studio for C-DiGit Blot Scanner.

### Serum Levels of Cytokines and Corticosterone, and Activities of CK and LDH

Serum levels (pg mL^−1^) of IL-1β, IL-6, IL-10, IL-15, and TNF-α were assessed using Luminex™ multiplex reagents according to the instructions of the manufacturer (Millipore, ST Charles, MO, USA). For cytokines’ measurement, an MILLIPLEX MAP Mouse Cytokine Panel—5 Plex (Millipore, cat. number MCYTOMAG-70K) was used. Samples were acquired on the Luminex MAP200 instrument and were analyzed using the 3.1 xPONENT System. Serum levels (μg dL^−1^) of corticosterone were determined after ethanol extraction by radioimmunoassay method, as previously described (34). Activities of CK and LDH (U L^−1^) were measured by commercial kits according to the instructions of the manu-facturer (LaborLab^®^, SP, Brazil).

### Histological Analysis

Extensor digitorum longus and soleus muscle samples were sliced at −24°C using a refrigerated cryostat microtome (Jung CM 1800, Leica, Germany). Longitudinal sections were obtained from each skeletal muscle sample (10 µm of thickness) and were stained with hematoxylin and eosin for morphological evaluation. The histological sections were photographically digitized (an increase of 40×) with a digital camera (Axiocam-HR, Carl Zeiss, Jena, Germany) mounted on a light microscope (AxioCam 2, Carl Zeiss, Jena, Germany).

### Immunofluorescence Analysis

The pattern of muscle fiber type composition, the AR and GR expressions were performed by immunofluorescence. EDL and soleus samples were sliced at −24°C using a refrigerated cryostat microtome (Jung CM 1800, Leica, Germany). Transverse sections from each skeletal muscle sample (10 µm of thickness) were obtained and fixed in Baker’s formalin-calcium for 30 min. After remaining 30 min at room temperature, these sections were immersed in TBS containing 0.1% Tween-20 for 10 min. The blocking was performed with 3% BSA for 1 h. Endogenous biotin binding was blocked with avidin/biotin kit (Vector Laboratory, Burlingame, CA, USA). The sections were incubated in TBS containing 0.1% Tween-20 overnight at 4°C with the primary antibodies MYH (SC12117), MYH2 (SC53094), Myosin IIa (SC71632), AR (SC820), phospho-AR (SC820P), GR (SC1004), and phospho-GR (SC1004P) from Santa Cruz Biotechnology (Santa Cruz, CA, USA) at dilution of 1:10 or 1:20.

After wash with TBS containing 0.1% Tween-20, all sections were incubated for 2 h at room temperature with Texas red goat anti-mouse IgG (Invitrogen, CA, USA; T862), Texas red-x goat antirabbit IgG (Invitrogen, CA, USA; T6391), FITC goat anti-mouse (Invitrogen, CA, USA; 626511), or FITC donkey antigoat (Santa Cruz Biotechnology, CA, USA; SC2024) at dilution of 1:20, and with 4′,6-diamidino-2-phenylindole, dihydrochloride (DAPI; Invitrogen, CA, USA; D1306) at dilution of 1:50. The sections were mounted with Vectashield Mounting Medium with DAPI (H-1200, Vector Laboratories, Burlingame, CA, USA) and examined using a confocal laser scanning microscope (CLSM, C2si, Nikon, Japan). Images were captured at 20× magnification. For muscle fiber type, the counting was performed in at least 250 fibers per animal, and the percentages of marked fibers were estimated. For AR and GR expressions, the counting was performed in at least 10 transverse sections of EDL and soleus per animal. ImageJ software version 1.5 for Windows was used for the quantification of the expressed nucleus.

### Statistical Analysis

Results were expressed as a mean ± standard error (SE). According to Shapiro–Wilk’s *W*-test, the data were normally distributed, and homogeneity of variances was confirmed by Levene’s test. Therefore, one-way analysis of variance was used to examine the effects of the OT protocols followed by Bonferroni’s *post hoc* test, when appropriate. All statistical analyses were two sided, and the significance level was set at *P* < 0.05. Statistical analyses were performed using STATISTICA 8.0 computer software (StatSoft^®^, Tulsa, OK, USA).

## Results

### Pro- and Anti-Inflammatory Cytokines in EDL, Soleus, and Serum

**Figure 1:**
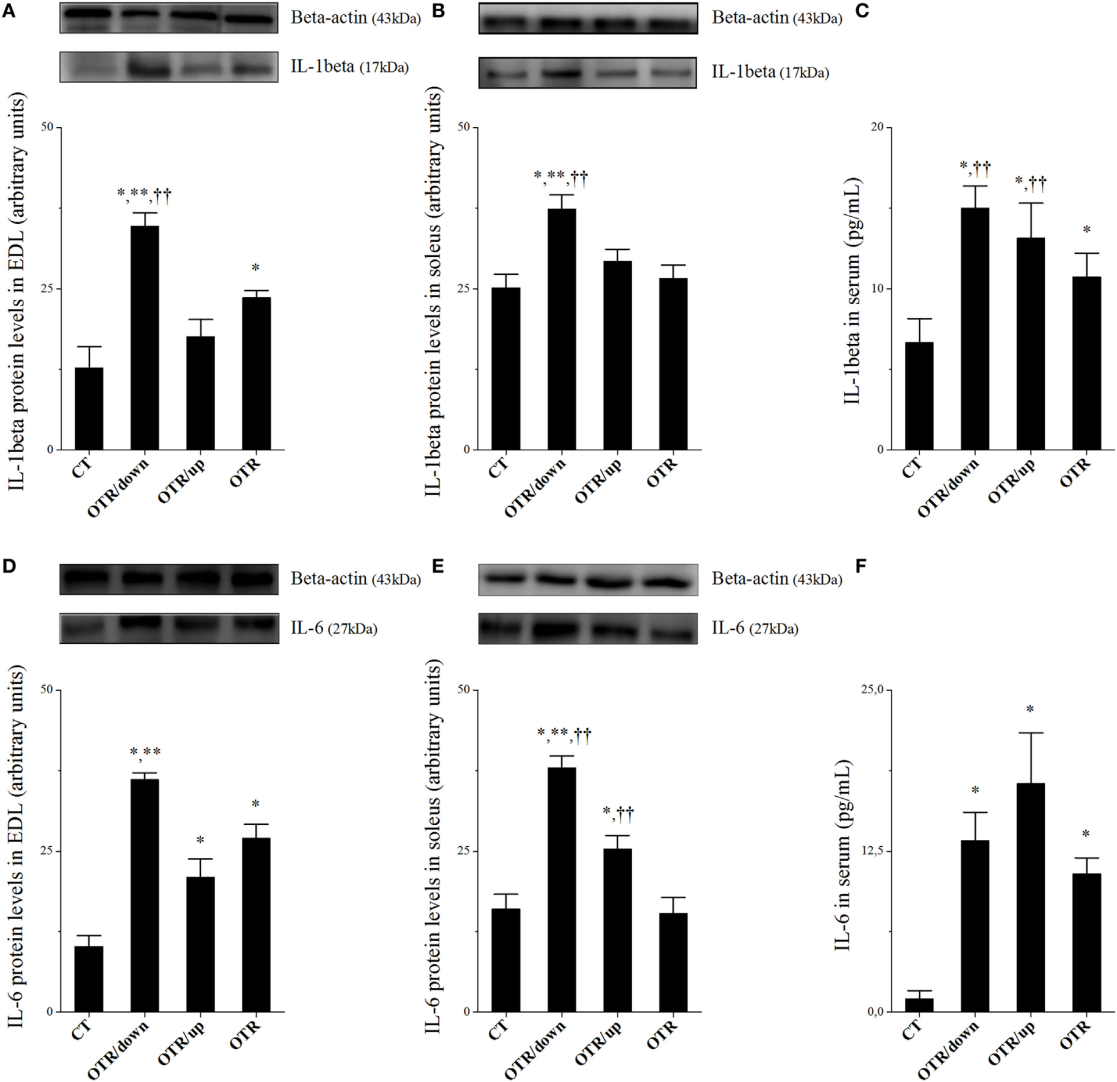

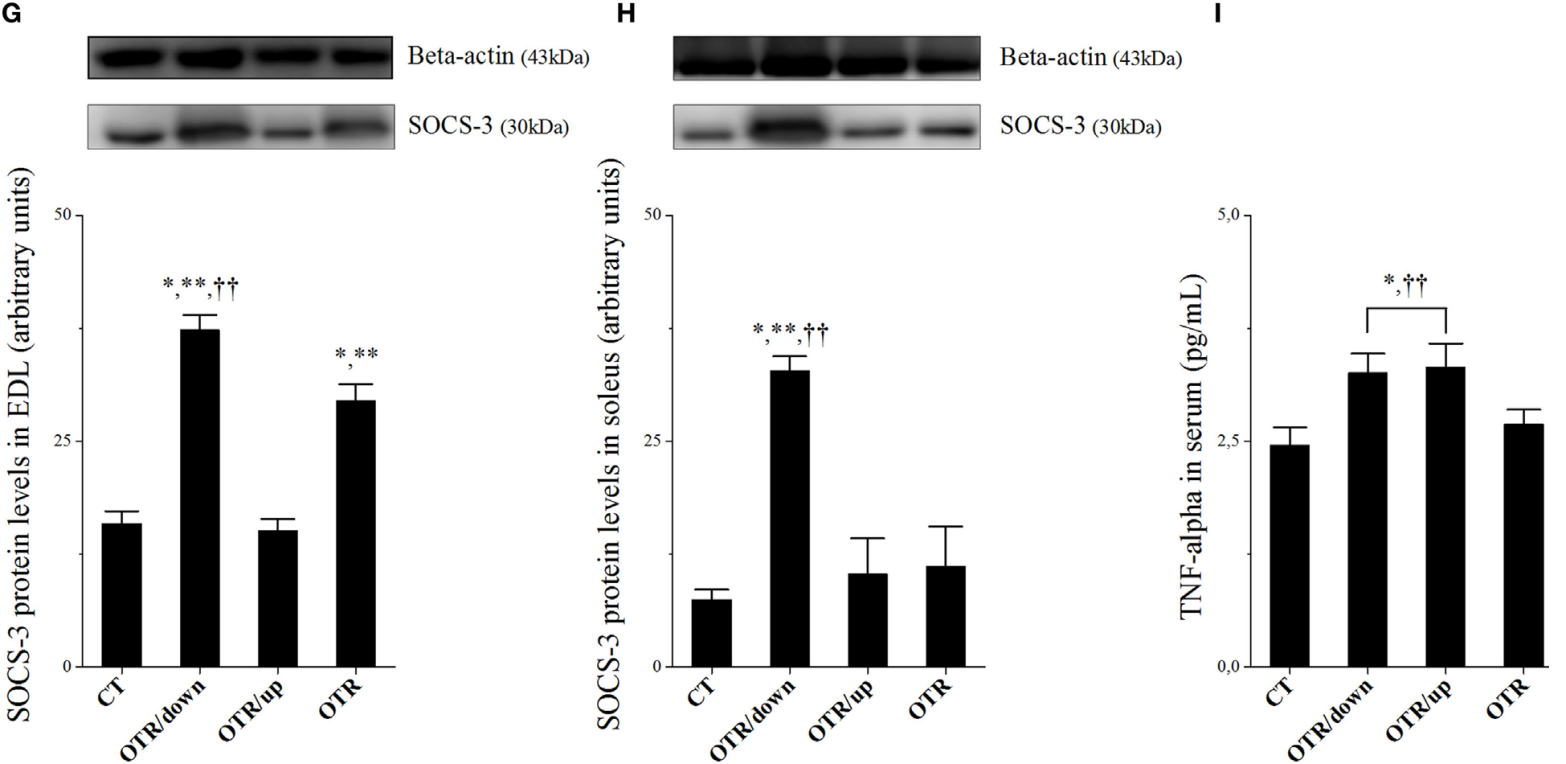
Protein levels (arbitrary units) of interleukin 1beta (IL-1β) in extensor digitorum longus (EDL) **(A)**, soleus **(B)**, and serum **(C)**; IL-6 in EDL **(D)**, soleus **(E)**, and serum **(F)**; SOCS-3 in EDL **(G)** and soleus **(H)**; tumor necrosis factor alpha (TNF-α) in serum **(I)**. Data correspond to mean ± standard error (SE) of *n* = 6. Control (CT): sedentary mice. OTR/down: overtrained by downhill running. OTR/up: overtrained by uphill running. OTR: overtrained by running without inclination. *P < 0.05 vs. CT; **P < 0.05 vs. OTR/up; ^††^*P* < 0.05 vs. OTR.

Protein levels of IL-1β and SOCS-3 in EDL and soleus for the OTR/down group were significantly higher than the other groups (Figures 1A,B,G,H, respectively). While protein levels of IL-1β in EDL for the OTR group were significantly higher compared to the CT group (Figure 1A), protein levels of SOCS-3 in EDL for the OTR group were significantly higher compared to the CT and OTR/up groups (Figure 1G). Figure 1C showed that IL-1β serum levels increased significantly after all OT protocols compared to the CT group. Also, IL-1β serum levels for the OTR/down group were significantly higher compared to the OTR group.

Protein levels of IL-6 in EDL increased significantly after all OT protocols compared to the CT group. Also, the OTR/down group presented significantly higher values compared to the OTR/up group (Figure 1D). While protein levels of IL-6 in the soleus for the OTR/down group were significantly higher than the other experimental groups, the OTR/up group presented significantly higher values compared to the CT and OTR groups (Figure 1E). Serum levels of IL-6 for all OT groups were significantly higher than the CT group (Figure 1F). Figure 1I shows that serum levels of TNF-α for the OTR/down and OTR/up groups were significantly higher compared to the CT and OTR groups.

**Figure 2:**
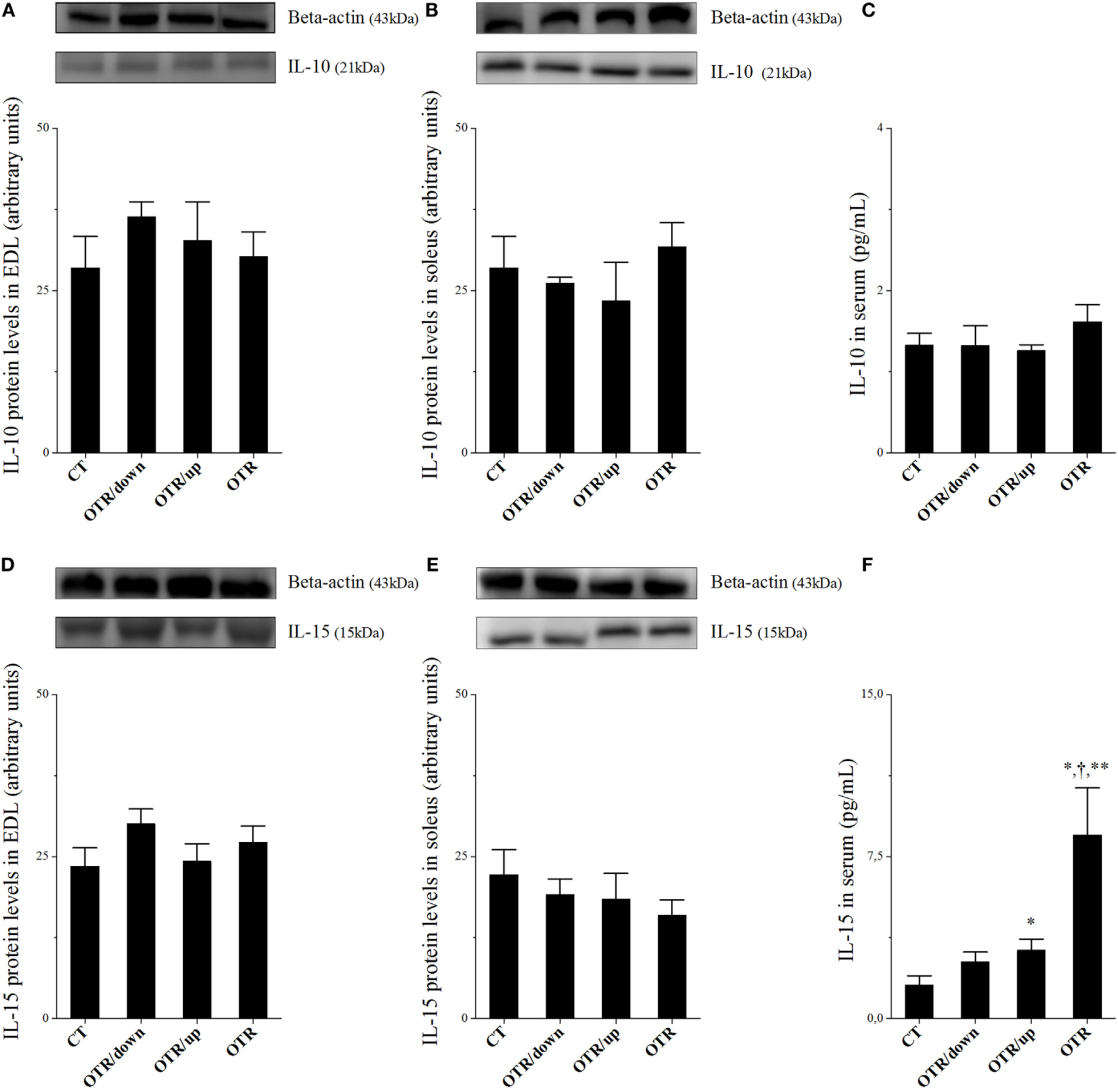
Protein levels (arbitrary units) of interleukin (IL)-10 in extensor digitorum longus (EDL) **(A)**, soleus **(B)**, and serum **(C)**; IL-15 in EDL **(D)**, soleus **(E)**, and serum **(F)**. Data correspond to mean ± standard error (SE) of *n* = 6. Control (CT): sedentary mice. OTR/down: overtrained by downhill running. OTR/up: overtrained by uphill running. OTR: overtrained by running without inclination. *P < 0.05 vs. CT; ^†^*P* < 0.05 vs. OTR/down; **P < 0.05 vs. OTR/up.

Protein levels of IL-10 in EDL, soleus, and serum, as well as protein levels of IL-15 in EDL and soleus were not different among the experimental groups (Figures 2A–E, respectively). While the OTR/up group presented significantly higher serum levels of IL-15 compared to the CT group, the OTR group presented significantly higher serum levels of IL-15 compared to all experimental groups (Figure 2F).

### Histological Analysis

**Figure 3:**
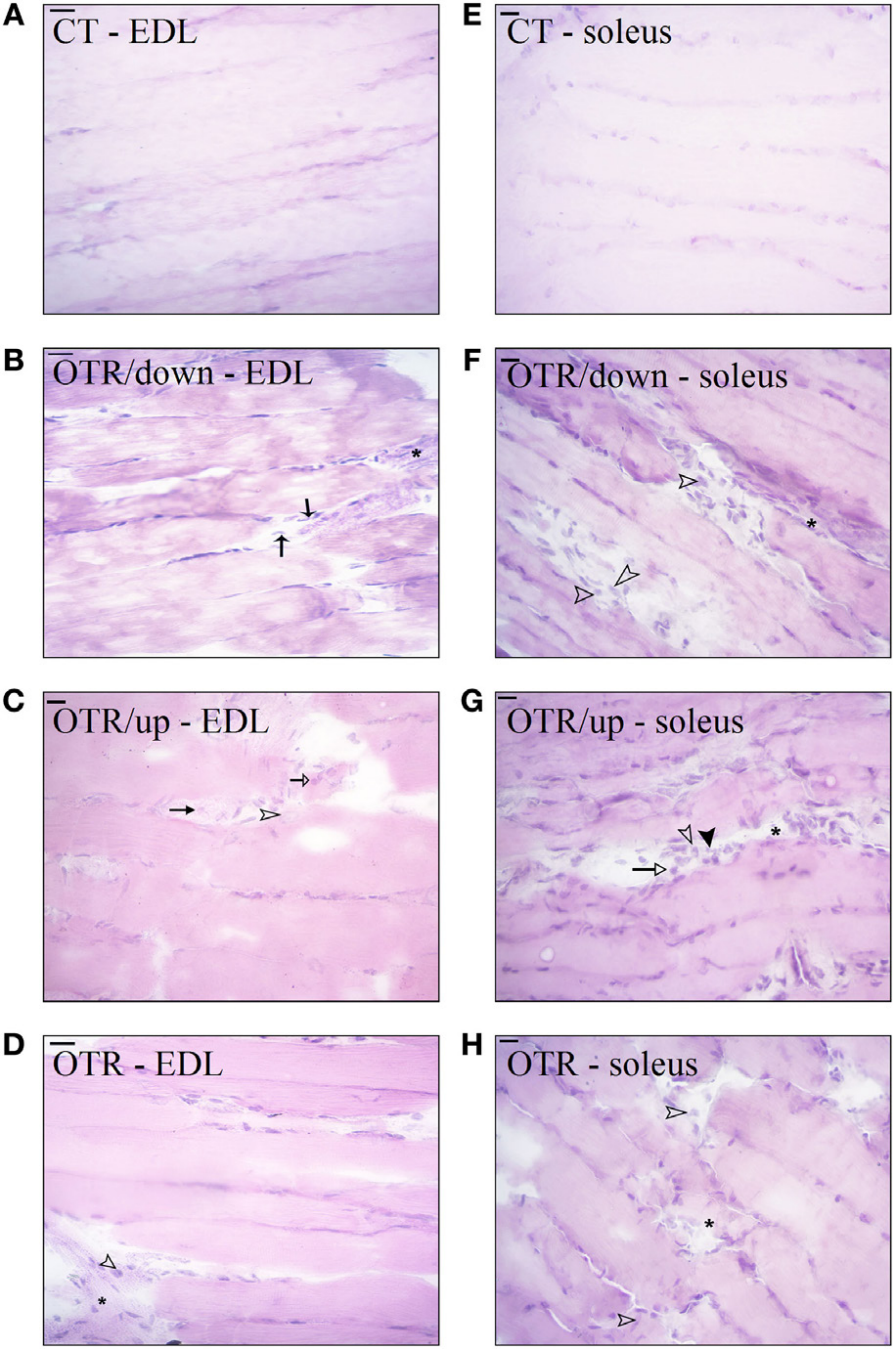
Histological analysis of extensor digitorum longus (EDL) and soleus longitudinal sections from control (CT) **(A,E)**, downhill running-based overtraining (OTR/down) **(B,F)**, OTR/up **(C,G)**, and OTR **(D,H)**. Data correspond to *n* = 6 mice. CT: sedentary mice. OTR/down: overtrained by downhill running. OTR/up: overtrained by uphill running. OTR: overtrained by running without inclination. *Micro-injuries with polymorphonuclear infiltrated. 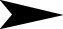 pyknotic leukocytes. 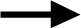 neutrophils. 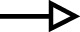 lymphocytes. 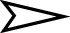 macrophages.

Hematoxylin and eosin stains from muscle samples showed that EDL and soleus longitudinal sections of CT presented normal-looking fibers (Figures 3A,E). EDL longitudinal sections of OTR/down and OTR, and soleus longitudinal sections of OTR/down, OTR/up, and OTR presented micro-injuries with polymorphonuclear infiltrated (Figures 3B–H, respectively). Figures 3B,C showed that EDL longitudinal sections of OTR/down and OTR/up presented infiltration of macrophages and neutrophils. EDL longitudinal sections of OTR and soleus longitudinal sections of OTR/down, OTR/up, and OTR presented infiltration of macrophages (Figures 3D,H, respectively). Finally, Figure 3G showed that soleus of OTR/up presented pyknotic leukocytes.

### Serum Levels of Corticosterone and Activities of CK and LDH at the End of Week 8

Corticosterone serum levels for the OTR group were significantly higher compared to the CT group. Activities of CK and LDH were not different among the experimental groups (Table 1).

**Table 1:** Responses of serum corticosterone (μg dL^−1^), and activities of creatine kinase (U L^−1^) and lactate dehydrogenase (U L^−1^) in the CT, OTR/down, OTR/up, and OTR groups.

	CT	OTR/down	OTR/up	OTR
Corticosterone (μg dL^−1^)	8.7 ± 1.3	9.6 ± 0.7	11.0 ± 1.4	14.2 ± 0.9*
Creatine kinase (U L^−1^)	1,130 ± 250	1,100 ± 270	1,280 ± 490	1,670 ± 530
Lactate dehydrogenase (U L^−1^)	137.7 ± 32.9	144.5 ± 33.5	168.8 ± 49.9	187.3 ± 34.2

Data correspond to the mean ± SE of n = 5–6 mice. CT: sedentary mice; OTR/down: overtrained by downhill running; OTR/up: overtrained by uphill running; OTR: overtrained by running without inclination. *P < 0.05 vs. CT group.

### Pattern of Muscle Fiber Type Composition

**Figure 4:**
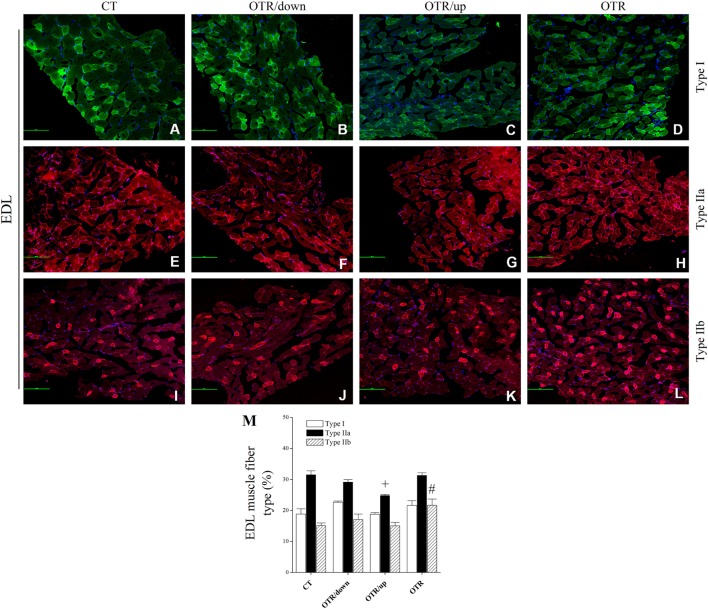
Immunofluorescence images of extensor digitorum longus (EDL) muscle fiber type composition from control (CT) [**(A)** type I, **(E)** type IIa, and **(I)** type IIb], downhill running-based overtraining (OTR/down) [**(B)** type I, **(F)** type IIa, and **(J)** type IIb], OTR/up [**(C)** type I, **(G)** type IIa, and **(K)** type IIb], and OTR [**(D)** type I, **(H)** type IIa, and **(L)** type IIb]. Percentage of type I, type IIa, and type IIb **(M)** fibers in EDL muscle. Data correspond to *n* = 4 mice. CT: sedentary mice. OTR/down: overtrained by downhill running. OTR/up: overtrained by uphill running. OTR: overtrained by running without inclination. ^+^*P* < 0.05 vs. CT, OTR/down, and OTR; ^#^*P* < 0.05 vs. CT and OTR/up.

**Figure 5:**
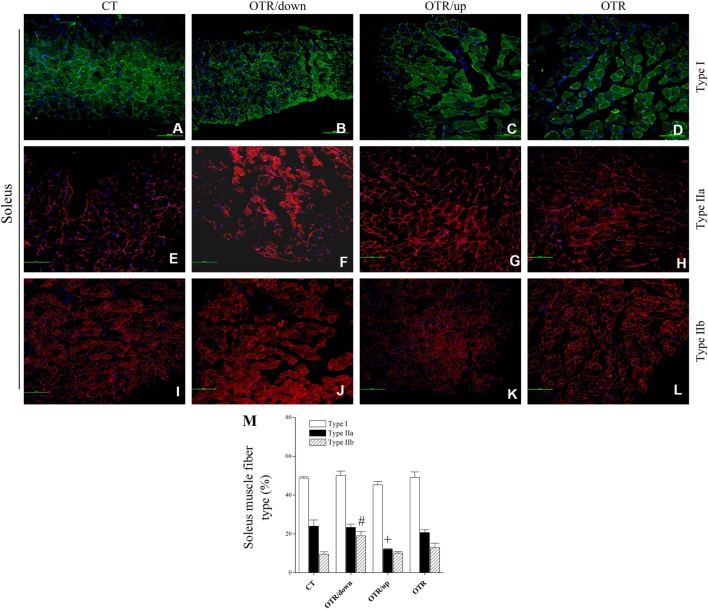
Representative immunofluorescence images of soleus muscle fiber type composition from control (CT) [**(A)** type I, **(E)** type IIa, and **(I)** type IIb], downhill running-based overtraining (OTR/down) [**(B)** type I, **(F)** type IIa, and **(J)** type IIb], OTR/up [**(C)** type I, **(G)** type IIa, and **(K)** type IIb], and OTR [**(D)** type I, **(H)** type IIa, and **(L)** type IIb]. Percentage of type I, type IIa, and type IIb **(M)** fibers in soleus muscle. Data correspond to *n* = 4 mice. CT: sedentary mice. OTR/down: overtrained by downhill running. OTR/up: overtrained by uphill running. OTR: overtrained by running without inclination. ^+^*P* < 0.05 vs. CT, OTR/down, and OTR; ^#^*P* < 0.05 vs. CT, OTR/up, and OTR.

Representative immunofluorescence images of muscle fiber type composition for the CT, OTR/down, OTR/up, and OTR groups are presented in the Figures 4A–D (i.e., type I of EDL), Figures 4E–H (i.e., type IIa of EDL), Figures 4I–L (i.e., type IIb of EDL), Figures 5A–D (i.e., type I of soleus), Figures 5E–H (i.e., type IIa of soleus), and Figures 5I–L (i.e., type IIb of soleus). The percentage of type I fibers of EDL and soleus was not different among the experimental groups (Figures 4M and 5M). The percentage of type IIa fibers of EDL and soleus for the OTR/up group was significantly lower compared to the CT, OTR/down, and OTR groups (Figures 4M and 5M). While the percentage of type IIb fibers of EDL for the OTR group was significantly higher compared to the CT and OTR/up groups (Figure 4M), the percentage of type IIb fibers of soleus for the OTR/down group was significantly higher compared to the other experimental groups (Figure 5M).

### AR and GR Expressions

**Figure 6:**
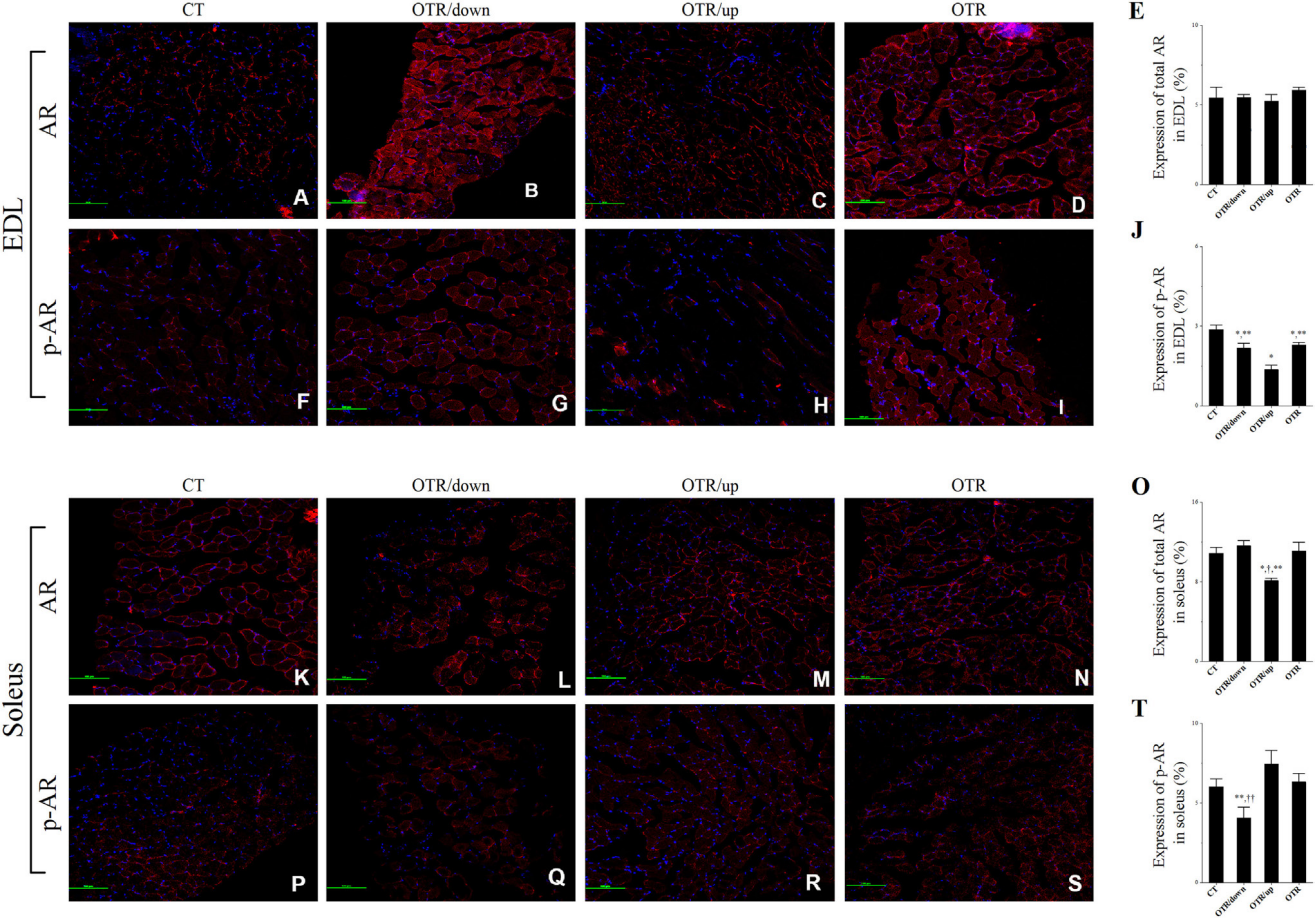
Representative immunofluorescence images of total and phosphorylated androgen receptor (AR) expressions in extensor digitorum longus (EDL) and soleus from control (CT) **(A,F,K,P)**, downhill running-based overtraining (OTR/down) **(B,G,L,Q)**, OTR/up **(C,H,M,R)**, and OTR **(D,I,N,S)**. Percentage of total and phosphorylated AR expressions in EDL **(E,J)** and soleus **(O,T)** muscles. Data correspond to *n* = 4 mice. CT: sedentary mice. OTR/down: overtrained by downhill running. OTR/up: overtrained by uphill running. OTR: overtrained by running without inclination. *P < 0.05 vs. CT; ^†^*P* < 0.05 vs. OTR/down; **P < 0.05 vs. OTR/up; ^††^*P* < 0.05 vs. OTR.

Representative immunofluorescence images of total and phosphorylated AR levels in EDL and soleus for the CT, OTR/down, OTR/up, and OTR groups are presented in the Figures 6A–D,F–I,K–N,P–S. The total AR expressions in EDL were not different among the experimental groups (Figure 6E). The three overtrained groups presented lower phosphorylated AR levels in EDL compared to the CT group. Also, the phosphorylated AR levels in EDL for the OTR/down and OTR were higher compared to the OTR/up group (Figure 6J). While the total AR expression in soleus for the OTR/up group was lower compared to the other experimental groups (Figure 6O), the phosphorylated AR level in soleus for the OTR/down group was lower compared to the other OT groups (Figure 6T).

**Figure 7:**
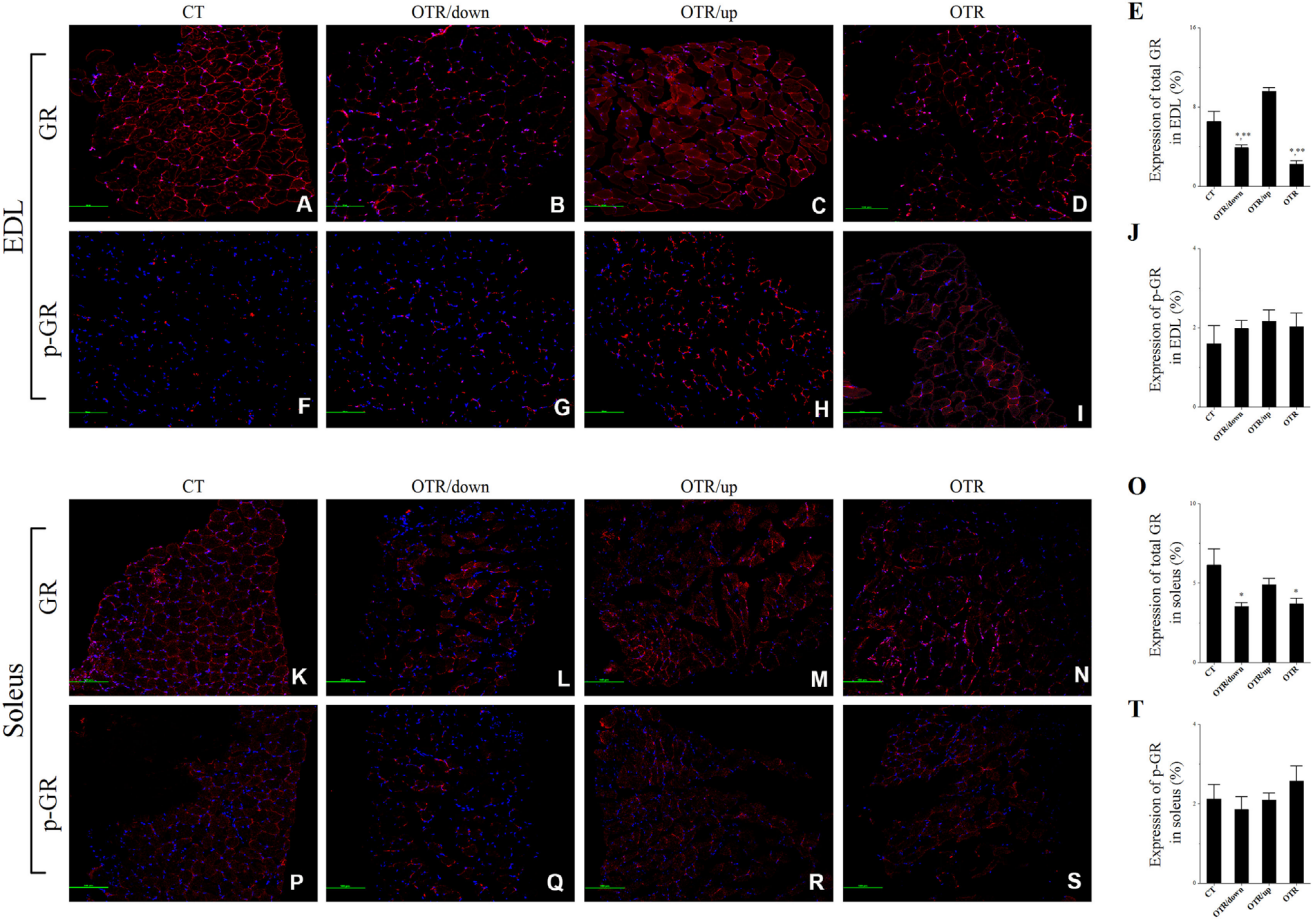
Representative immunofluorescence images of total and phosphorylated glucocorticoid receptor (GR) expressions in extensor digitorum longus (EDL) and soleus from control (CT) **(A,F,K,P)**, downhill running-based overtraining (OTR/down) **(B,G,L,Q)**, OTR/up **(C,H,M,R)**, and OTR **(D,I,N,S)**. Percentage of total and phosphorylated GR expressions in EDL **(E,J)** and soleus **(O,T)** muscles. Data correspond to *n* = 4 mice. CT: sedentary mice. OTR/down: overtrained by downhill running. OTR/up: overtrained by uphill running. OTR: overtrained by running without inclination. *P < 0.05 vs. CT; **P < 0.05 vs. OTR/up.

Representative immunofluorescence images of total and phosphorylated GR levels in EDL and soleus for the CT, OTR/down, OTR/up, and OTR groups are presented in the Figures 7A–D,F–I,K–N,P–S. The total GR expressions in EDL for the OTR/down and OTR groups were lower compared to the CT and OTR/up groups (Figure 7E). The phosphorylated GR levels in EDL were not different among the experimental groups (Figure 7J). The total GR expressions in soleus for the OTR/down and OTR groups were lower compared to the CT group (Figure 7O). The phosphorylated GR levels in soleus were not different among the experimental groups (Figure 7T).

## Discussion

The main findings of the present investigation were (a) in the EDL, the OTR/down and OTR groups increased all analyzed proinflammatory cytokines and exhibited microinjuries with polymorphonuclear infiltration; (b) in the soleus, while the OTR/down group increased all analyzed proinflammatory cytokines, the OTR/up group only increased IL-6. Also, the three OT groups presented micro-injuries with polymorphonuclear infiltration; (c) in the circulating cytokines and corticosterone concentrations, while the OTR/down and OTR/up protocols led to similar increases of IL-1β, IL-6, and TNF-α, the OTR protocol increased IL-1β, IL-6, IL-15, and corticosterone; (d) except for the type I fibers of EDL and soleus, the three OT groups presented different patterns of muscle fiber type composition; (e) the total and phosphorylated AR levels in the soleus, and the total GR expressions in EDL and soleus were differentially modulated by the OT protocols.

The current EDL and soleus contents of IL-6 and SOCS-3 for the OTR/down group observed in the present study reproduced our previous findings (5, 21). Recently, we demonstrated that this specific OT model also increased IL-1β and TNF-α (35) in both skeletal muscle samples. Furthermore, histological analysis revealed microinjuries with polymorphonuclear infiltration in the EDL and soleus, macrophage and neutrophil infiltration in the EDL, and macrophage infiltration in the soleus. These results are in accordance with Fielding et al. (36) who verified that eccentric exercise-induced skeletal muscle damage was associated with neutrophil infiltration and IL-1β accumulation.

Considering the crosstalk between inflammation and endoplasmic reticulum (ER) stress (37), Pereira et al. (10) recently verified that the OTR/down protocol led to ER stress in the EDL and soleus. Because most of the analyzed ER stress proteins were not normalized after a 2-week recovery period and based on the investigation of Rayavarapu et al. (38), the authors suggested a possible pathological condition of ER stress in both skeletal muscle types (10). The present findings of elevated serum levels of IL-1β, IL-6, and TNF-α reinforced the low-grade chronic inflammation state linked to OTR/down (5, 7). Although IL-6 has both anti- and proinflammatory properties (39) and its systemic levels increased after moderate exercise (40), the response of this specific cytokine to sepsis or regular exercise differed from the increase in TNF-α (41), which sustained the OTR/down-induced inflammatory status.

Compared to the other OT protocols, the OTR/up model led to a lower modulation of intramuscular cytokine concentrations. Indeed, only the soleus contents of IL-6 and TNF-α (35) were upregulated after this OT protocol. These results probably contributed to the increase of some ER stress proteins in this particular skeletal muscle sample (10). Herein, soleus samples displayed microinjuries with polymorphonuclear infiltration. Similarly to the OTR/down model, the OTR/up protocol increased the serum levels of IL-1β, IL-6, and TNF-α, which demonstrated that this specific protocol also led to systemic inflammation. The elevated levels of soleus IL-6 and TNF-α (35) and of serum IL-1β, IL-6, and TNF-α observed in the OTR/up group highlight the damaging effects of the relation between high-load exercise sessions and inadequate recovery because these cytokines usually do not increase in response to training-induced positive adaptations (5, 42).

Although high muscle levels of proinflammatory cytokines in response to downhill running are well described (5, 43–45), we showed that a running protocol with the same external load, but performed without inclination, also upregulated IL-1β, IL-6, and SOCS-3 in EDL. Recently, we demonstrated that this OT model increased the EDL and soleus contents of TNF-α (35). Furthermore, both muscle samples presented micro-injuries with polymorphonuclear and macrophage infiltration. As observed in the OTR/down group, serum levels of IL-1β and IL-6 also increased after OTR, suggesting the inflammatory status induced by this specific OT protocol. The lack of an increase in serum TNF-α in response to OTR may be related to the expressive increase in serum IL-15, because Marzetti et al. (20) verified that these cytokines were negatively correlated in aging rodent muscles.

Even performed with the same relative intensity (7–11), the high content of TNF-α in the soleus was the unique common intramuscular alteration between the OT protocols (35). Because TNF-α induces hyperalgesia (46) and its signaling blockade reduces skeletal muscle contraction dysfunction after eccentric exercise (47), this cytokine may play a pivotal role in the performance declines observed in these groups (7–11). Thus, further research should attempt to inhibit TNF-α after OT protocols to test our previous hypothesis. Furthermore, macrophage infiltration also occurred in the soleus of the three OT protocols and may be related to tissue repair promotion and the recovery of muscle metabolism homeostasis (48).

It is known IL-6 activates the HPA axis during local inflammation, which increases the corticosterone levels (26). Although our OT models displayed elevated levels of serum IL-6, only the OTR group presented increased levels of serum corticosterone. In accordance, Lira et al. (49) also verified that overtrained rats increased the serum levels of corticosterone 24 h after their last session of exercise. The serum levels of CK and LDH of our overtrained groups were analyzed 36 h after the last performance test and did not present significant alterations. These classical markers of muscle damage were not sensitive to another OT model developed for Wistar rats (50–52).

Herein, for the first time, we described the pattern of muscle fiber type composition after the three OT models linked to NFOR state. Interestingly, the OTR/up protocol decreased the percentage of type IIa muscle fibers in both EDL and soleus samples compared to the other experimental groups. This may be considered a negative adaptation related to the uphill running excessive training since other authors did not observe significant changes of type IIa muscle fibers after uphill running moderate training (53, 54).

The OTR protocol increased the percentage of type IIb fibers of EDL compared to the CT and OTR/up groups. Previously, Gandra et al. (55) showed that Wistar rats classified in the functional overreaching (FOR) state, which is characterized by a performance improvement in response to days of recovery after an OT period (2), decreased the myosin heavy chain (MyHC) IIb and increased the MyHC IIa of red gastrocnemius, suggesting a shift toward a more efficient fiber-type composition for endurance exercise. To investigate the white gastrocnemius of hypertensive Dahl/SS rats, Holloway et al. (56) verified that endurance training reduced type IIb fibers, while high-intensity training increased type IIb fibers.

The OTR/down protocol decreased the percentage of type IIb fibers of soleus compared to the other experimental groups. In accordance, Azad et al. (57) observed a reduction in type IIb fibers of soleus after an acute eccentric exercise protocol (i.e., 16 m min^−1^ on − 16° slope for 3 consecutive days, which includes 18 sets of 5 min with a rest interval of 2 min in between). The different patterns of muscle fiber type composition observed in the current overtrained groups were probably related to their specific treadmill inclination since the OT protocols were performed with the same external load. Future investigations should analyze some fundamental metabolism proteins such as the peroxisome proliferator-activated receptor-gamma coactivator 1alpha (PGC-1α) to provide indications of a metabolic shift in the overtrained muscles (i.e., from oxidative to glycolytic pattern).

Recently, Yin et al. (58) described an increase of total AR expression in gastrocnemius and no significant changes in soleus after a running OT protocol. However, these authors also showed that their overtrained group displayed a higher time to exhaustion compared to the sedentary rats. Knowing that a decrease or stagnation of performance is the only marker for the diagnosis of NFOR (1, 2, 59, 60), we consider that they evaluated rats in the FOR state. Therefore, to our knowledge, this is the first study describing the responses of total and phosphorylated AR and GR in skeletal muscle samples to three different OT models linked to the NFOR state.

Because total-limb maximal grip strength was decreased in satellite cell-specific AR-knockout mice (61), we considered that the lower total AR expressions in the soleus and the lower phosphorylated AR levels in EDL and soleus were probably related to the reduced levels of the grip strength test that were previously observed after these three OT protocols (7, 9, 32, 35). Also, the suppression of AR reduces the exercise-induced skeletal muscle hypertrophy (62). Interestingly, da Rocha et al. (9) verified that the OTR/down protocol inhibited the skeletal muscle hypertrophy with concomitant signs of atrophy in the EDL muscle sample.

The role of GR in skeletal muscle atrophy and weakness is well documented in the literature (27). Also, Willoughby et al. (63) verified that eccentric exercise decreased the strength and increased the GR expression in human vastus lateralis muscle. Because our models of OT reduced the grip strength performance (7, 9, 32, 35), we first expected an upregulation of total and phosphorylated GR levels in EDL and soleus of the overtrained mice. While total GR expressions were lower for the OTR/down and OTR groups, phosphorylated GR levels were not different among the experimental groups. Coutinho et al. (64) suggested that the exercise-induced reduction of GR expression may act as a protective mechanism from the damaging effects of continuous increases in glucocorticoids. Considering the corticosterone levels were higher in the OTR group, we consider the previous hypothesis (64) may be partially explained by our current findings.

**Figure 8:**
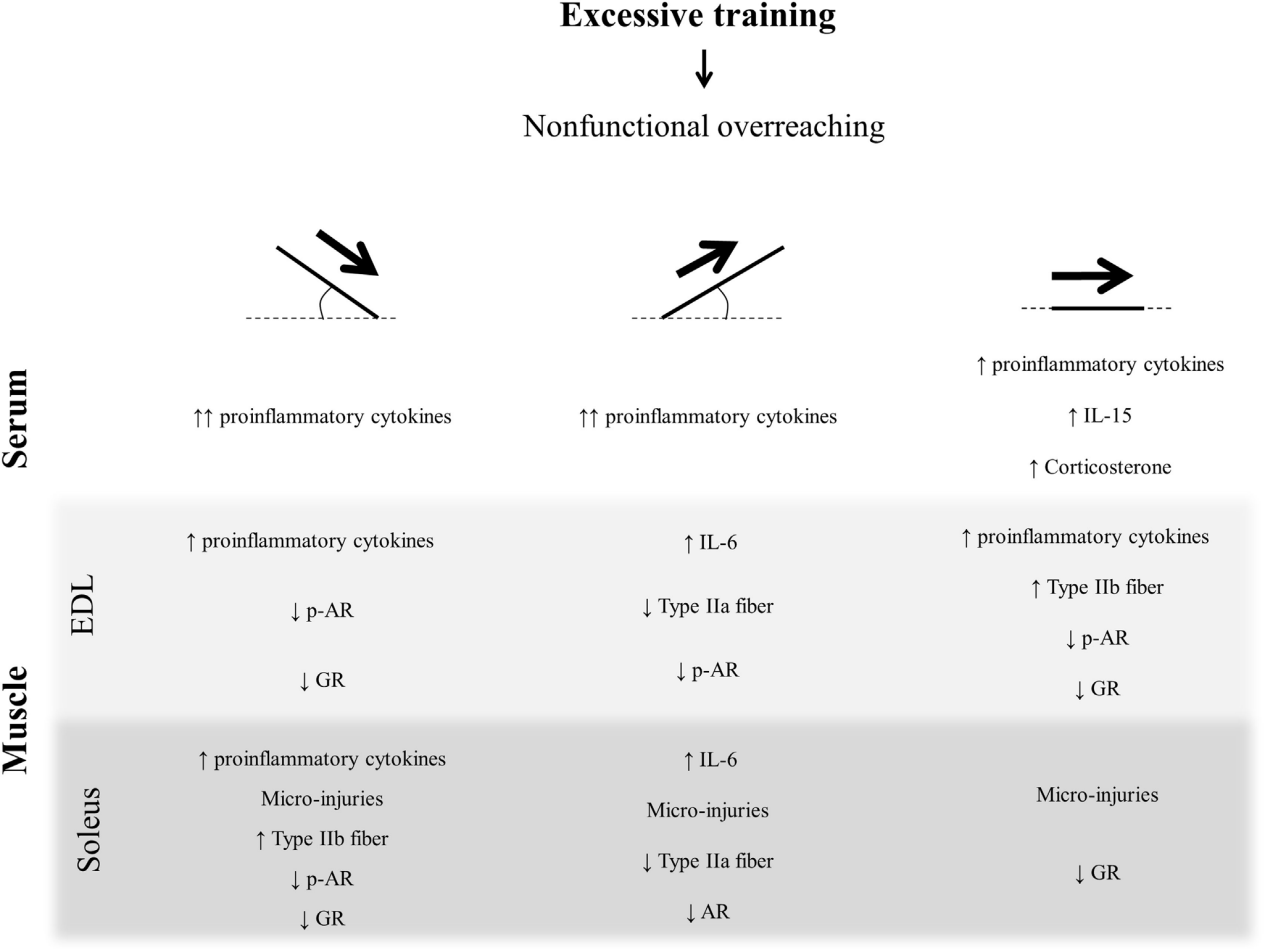
Schematic model summarizing the present findings.

In conclusion, the three OT protocols were linked to a proinflammatory status and led to skeletal muscle tissue injury, which may be related to the NFOR state-induced performance reduction. Indeed, the proinflammatory cytokines were more sensitive for the OTR/down than for OTR/up and OTR. The OTR protocol for EDL and the OTR/down protocol for soleus increased the MHC IIb composition, which may be related to a metabolic change from oxidative to glycolytic pattern. Although the three OT groups presented high serum levels of IL-6, only the OTR model increased the serum levels of corticosterone. The AR and GR expressions in EDL and soleus were not similar among the overtrained groups, emphasizing the specificity of the predominating muscle contraction type in the different degrees of treadmill inclination. The present data are summarized in Figure 8 and highlight the importance of surveillance of athletes who are at risk to develop NFOR state, which culminates in performance stagnation or reduction.

## Ethics Statement

This study was carried out in accordance with the recommendations of the Brazilian College of Animal Experimentation (COBEA). The protocol was approved by the Ethics Committee of the University of Sao Paulo (ID 14.1.873.53.0).

## Author Contributions

Conceived and designed the experiments: AR, BP, and AS. Performed the experiments: AR, BP, GT, AP, FF, FL, and LE. Analyzed the data: AR, BP, FF, LE, and FL. Contributed reagents/materials/analysis tools: GT, FF, LE, FL, JP, DC, ER, LM, RM, EF, and AS. Wrote the article: AR and AS. Revised the work critically for important intellectual content: AP, FF, LE, FL, JP, DC, ER, LM, RM, and EF. Final approval of the version to be published: BP, GT, AP, FF, LE, FL, JP, DC, ER, LM, RM, and EF.

## Conflict of Interest Statement

The authors declare that the research was conducted in the absence of any commercial or financial relationships that could be construed as a potential conflict of interest.
